# The Role of Digital Health Technology Interventions in the Prevention of Type 2 Diabetes Mellitus: A Systematic Review

**DOI:** 10.1177/11795514241246419

**Published:** 2024-05-21

**Authors:** Vivien Nguyen, Paige Ara, David Simmons, Uchechukwu Levi Osuagwu

**Affiliations:** 1 School of Medicine, Western Sydney University, Campbelltown, NSW, Australia; 2 Translational Health Research Institute, Western Sydney University, NSW, Australia; 3 Bathurst Rural Clinical School (BRCS), School of Medicine, Western Sydney University, Bathurs, NSW, Australia

**Keywords:** Digital health, review, diabetes, hemoglobin A1c, health technology, health intervention

## Abstract

**Objectives:**

Diabetes in the 21st century presents one of the greatest burdens of disease on the global population. Digitally mediated interventions have become imperative in alleviating this disease epidemic. We aimed to systematically review randomized controlled trials (RCTs) on different health technologies for preventing Type 2 diabetes mellitus, and their efficacy in decreasing diabetes risk-related outcomes in at-risk patients in comparison to standard care.

**Methods:**

Five electronic databases were searched between October 2021 and December 2022. Studies including digital health technology interventions used for preventing diabetes development by reducing diabetes risk-related outcomes in at-risk adults (⩾18 years) were identified. Data on glycemic levels, incidence of T2DM, weight, and intervention descriptions were extracted, and the risk of bias (ROB) was assessed.

**Results:**

Nine studies met the inclusion criteria and 5 studies (56%) achieved clinically significant outcomes in at least one of the following: decreased weight (22%), glycemic levels (22%), or incidence of T2DM (11%). Two of the 3 (67%) computer-based interventions effectively reduced the HbA1c levels and mean weight of their study population, and 3 of 6 (50%) mobile based interventions (text messages, mobile app, and telehealth) decreased the incidence of T2DM and HbA1c levels. Four studies each had an overall low ROB and one had a high ROB due to attrition.

**Conclusion:**

Preliminary evidence identified in our review demonstrated that health technologies for diabetes prevention are effective for improving diabetes risk-related outcomes. Future research into digital technology protocol and studies of longer duration and more diverse populations are needed for clinical feasibility.

## Plain Language Summary

Role of Digital technology in Diabetes prevention

In the 21st century, diabetes has become a major health challenge globally. To address this, we conducted a systematic review, looking at studies that used digital interventions to prevent Type 2 diabetes mellitus (T2DM) and how effective they are compared to standard care. In this study, we searched electronic databases from October 2021 to December 2022 and identified 9 studies that met our criteria. These studies focused on adults aged 18 and above who were at risk of developing diabetes. We looked at outcomes like glucose concentrations, T2DM incidence, and weight, and assessed the risk of bias in each study. The results showed that more than half of the studies showed significant outcomes. For instance, some interventions led to decreased weight, lower glucose concentrations, or reduced incidence of T2DM. Computer-based interventions and mobile-based interventions (including text messages, mobile apps, and telehealth) were particularly effective in improving these outcomes. In conclusion, our review suggests that digital health technologies can be effective in preventing diabetes and improving related outcomes. However, we note that more research is needed, especially looking at diverse populations and longer study durations, to confirm the clinical feasibility of these digital interventions for diabetes prevention. This is a promising step forward in using technology to tackle the growing diabetes epidemic, offering new ways to support individuals at risk and improve their health outcomes.

## Introduction

Type 2 Diabetes Mellitus (T2DM) is defined as a metabolic disease involving defective insulin action.^1^ The resulting hyperglycemia places individuals at high risk for all-cause mortality by health expenditure of around 966 billion USD.^2^ An estimated 50% of diabetes cases globally remain undetected or undiagnosed^3^ and over half of diabetes’ economic cost lies in its treatment.^2^ Diabetes prevention programs (DPPs) are increasingly crucial in mediating this exponential disease epidemic. With healthcare systems’ gradual digitalization, the mediation of DPPs through technological platforms can aid in translating and disseminating these preventative interventions into clinical and public health settings.

Diabetes prevention is traditionally grounded in behavioral change theory, where lifestyle modifications are normalized through educational curriculum, health coaching and peer support in individual and group settings.^4^ Landmark clinical trials prove that intensive lifestyle interventions targeting physical activity and healthy diets are effective in reducing the progression from prediabetes to diabetes by 58% in comparison to pharmacological interventions.^5-8^ They yield long-term results at follow-up with a T2DM reduction of up to 43% that persists for decades for particular populations,^5,6^ but with the growing global incidence of diabetes, this evidence requires translation into a multifaceted systems approach.^9^ In a public health setting, literature indicates the need to develop local health economies that facilitate communication and accessibility between the different levels of health care.^9^ At the patient level, barriers to accessibility and adherence need to be overcome.^1,10^ At the clinical practice level, the increasing prevalence of diabetes-related complications^9^ requires a shift of patient management from episodic primary physician checks to a chronic care model.^11^ At an administrative level, the economic burden on public health systems can be ameliorated by socio-structural change via health policies, health education^12^ and the development of supportive community environments.^13^

Digital health technology refers to the use of digital and information technologies, such as mobile devices, wearables, health information systems, software applications, and telehealth services, to improve the delivery and management of healthcare.^14,15^ It covers a wide range of tools and solutions crafted to improve the efficiency, accessibility, and quality of healthcare services while empowering individuals to actively engage in their health management. The origins of digital health technology can be traced back to the integration of computers into healthcare settings during the mid-20th century. Initially used for administrative tasks, these technologies gradually expanded to encompass clinical and patient care.^16^ The rapid evolution of digital health technology was further fueled by the advent of the internet and the widespread use of personal computing devices. Over the past few decades, there has been a notable surge in the development and acceptance of digital health solutions. The widespread adoption of smartphones, wearable devices, and the availability of high-speed internet have been pivotal factors contributing to this growth.^17^ Digital health technologies now encompass a diverse array of applications, including electronic health records (EHRs), telemedicine, mobile health apps, remote patient monitoring, health informatics, and the utilization of artificial intelligence for healthcare analytics.^18,19^

Herman^20^ identified the key shared features accounting for the success of major DDP trials. These include individualization of patient interventions, flexibility between group and individual settings, a mixture of physical and behavioral activities and an “extensive network of training, feedback and clinical support.”^1^ Evidence indicates that health technologies fulfill all these criteria by extending diabetes prevention from clinical settings to creating an individual’s daily health-protective environment.^10^ Health technologies act as intermediary platforms that transfer information between patient and provider whilst normalizing behavioral interventions and lifestyle modifications in the patients’ surroundings and communities.

Regarding computer-based interventions, there is little evidence of its impact on diabetes prevention as software focuses on known diabetes and population management between various health providers, remote patient monitoring and diabetes self-management.^21^ A Cochrane meta-analysis comparing 16 computer-mediated diabetes education programs demonstrated a small decrease in HbA1c in patients diagnosed with T2DM and has potential in translation to preventative interventions.^22^

There is also a paucity of data on the efficacy of digital health technology in diabetes prevention. Past studies that showed evidence of positive correlations between digitally translated DPPs and improved diabetes risk-related outcomes have been limited by their short duration (5-24 months) and the lack of long-term follow-up. These affect the predictive validity of results by not accounting for fluctuating weight and glycemic levels. The heterogeneity of study location and participant demographic and high variability in program procedure affects the feasibility of public health implementation and scalability as study outcomes may not be generalizable to other national or global contexts. Due to these research gaps, this paper aims to systematically review and compare current evidence about digitalized DPPs’ efficacy in reducing the incidence of diabetes and identify any shared features that may assist in optimizing current interventions and improving health outcomes for at-risk individuals.

## Methods

This systematic review was conducted using guidelines from the Cochrane Handbook for Systematic Reviews of Interventions^23^ and the protocol was registered with the International Prospective Register of Systematic Reviews PROSPERO (#: CRD42022344100).

### Search strategy

A systematic literature search was conducted using 5 electronic databases (PubMed, EBSCO, Ovid Medline, EMBASE and Cochrane Library) in October 2022 with a search strategy structured using the PICO tool^24^ consisting of related keywords and medical subject headings (MeSH) as seen below:

- Population: T2DM, prediabet*
- Intervention: Technology health, digital technology, biomedical technology
- Comparison: standard treatment, control
- Outcomes: decrease* risk, weight loss, decreas* BMI, decreas* blood glucose, decreas* hyperglycemia, improv* glycemic control, decreas* waist circumference

Boolean search terms “OR” and “AND” enforced a distinction between specified and generalized terms. Filters limiting peer-reviewed journal articles to randomized controlled trials (RCTs) and study recency to the last 10 years were applied. The final search strategy was as follows: (((MM T2DM OR prediabet*[MeSH Major Topic]) AND (standard treatment OR control)) AND (MM Technology, health OR digital technology OR biomedical technology)) AND (decreas* risk OR weight loss OR decreas* BMI OR decreas* blood glucose OR decreas* hyperglycemia OR improv* glycemic control OR decreas* waist circumference). The same search strategy was replicated across the various databases.

### Eligibility criteria

Titles and abstracts of studies were screened by 2 independent reviewers (VN, PA) for eligibility. Results were compared to finalize selection and any disagreements were resolved through discussion. Studies were eligible for inclusion if they: (i) assessed adult (>18 years) populations who were at risk of developing diabetes; (ii) involved technology-based interventions which could be fully digitally-mediated or delivered in a mixed digital and face-to-face format; (iii) involved interventions explicitly aimed at reducing the risk of diabetes development; (iv) had outcome features related to body weight, glycemic levels or T2DM incidence (decreased BMI, decreased waist circumference, weight loss percentage, decreased fasting blood glucose or HbA1c); (v) in the RCT study format; and (vi) are available in English with full text.

Exclusion criteria included: (i) studies involving populations with diabetes diagnoses; (ii) interventions with no digital technology component; and (iii) interventions aimed at managing existing diabetes rather than preventing diabetes incidence.

### Data extraction

Due to the small scale of the systematic review (2 reviewers and less than 20 studies), data was stored using standard data extraction templates on Google Forms based on Cochrane guidelines.^23^ Data was extracted from all studies by 1 reviewer (VN) then a second reviewer (PG) independently repeated extraction on a random 20% of selected studies to confirm accuracy. Disagreements were resolved with discussion. Data about primary outcomes related to reducing diabetes risk were sought for extraction to compare intervention efficacy. Studies were grouped by these primary outcomes (decreased BMI, weight, glycemic levels, and T2DM incidence) and the mean and standard deviation of baseline and end-of-study results were tabulated with *P*-values and confidence intervals, where available, to compare statistical significance. Study variables extracted include the following intervention characteristics: author, year of publication, population sample size, study design, country of study, the study’s definition of “at-risk” and intervention characteristics, primary outcome unit of measurement and study duration. Narrative synthesis was chosen to summarize these intervention characteristics and outcomes due to clinical heterogeneity (population baseline characteristics and intervention methods) and methodological heterogeneity.

### Assessing bias

The quality of selected studies was assessed for risk of bias (RoB) using the Cochrane Collaboration Tool^25^ by 1 reviewer (VN). A random 20% of studies were screened by a second reviewer (PG) to confirm reliability. Disagreements were resolved with discussion. Studies were rated either “low risk,” “some concerns” or “high risk” over 5 different domains. They were deemed “low risk” if they scored “low risk” on all domains or if the domains of “some concern” were highly unlikely to affect study results and deemed “high risk” if they scored “high risk” in at least 1 domain. Results were presented in Robvis^26^ traffic light and weighted bar plots.

Quality of evidence was assessed using the GRADE scale^27^ where reviewers’ confidence in the similarity of the true effect and the estimated effect were graded by 4 levels of certainty—“high,” “moderate,” “low,” and “very low.”

## Results

**Figure 1.**
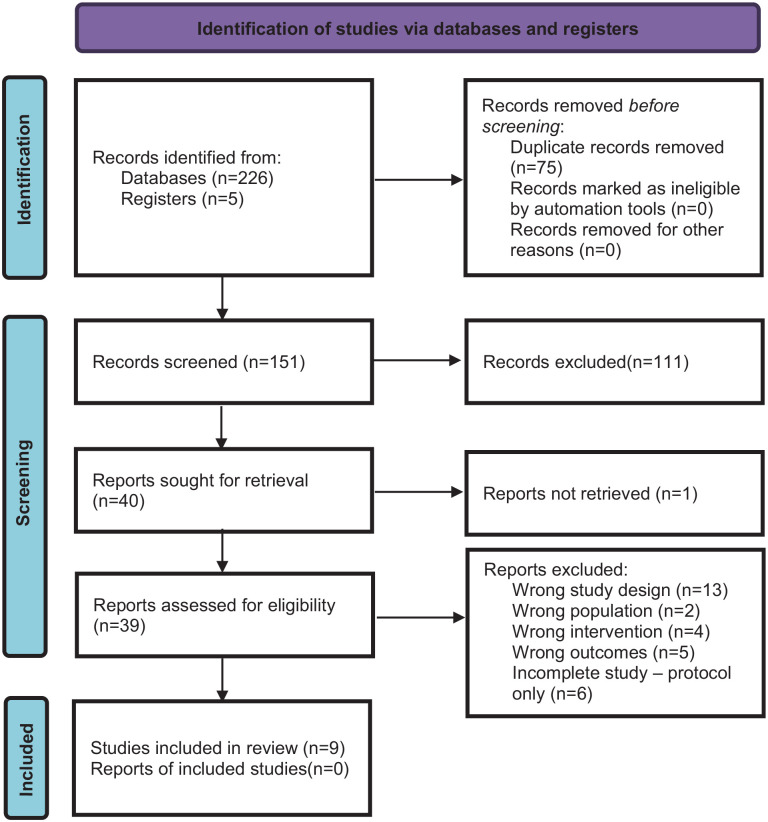
Flowchart of study selection.

Reporting of the study findings followed the Prisma protocol as shown in the checklist (see Supplemental Table 1). The PRISMA flow diagram^28^ shown in Figure 1 details the process of study selection and reasons for exclusion. The initial literature search identified 226 articles and after duplicates were removed 151 publications were screened by title and abstract. Forty studies were sought for retrieval and 1 study was not available on public databases. Thirty-nine studies from the following databases, PubMed (8), Ebsco (3) Ovid Medline (14), Cochrane (6), and other sources (8) underwent double screening and were assessed for eligibility after which 9 studies were included in this systematic review.

The basic characteristics of the reviewed studies are summarized in Table 1. Four of the studies that were included in the full-text review were from PubMed, 3 from Cochrane, and 2 from Medline libraries. Many of the studies (n = 44.4%) were conducted in America^29-33^ with a single study site each in New Zealand,^34^ India,^35^ Hong Kong,^36^ Bangladesh,^37^ and 1 jointly conducted in the UK and India.^31^ Eight out of 9 studies were parallel 2-arm RCTs^29-37^ while 1 study was a 3-arm cluster RCT.^37^ Six studies recruited participants from primary healthcare settings^29-34^, 3 studies recruited from the workplace,^32,35,36^ and 1 from the community.^37^

**Table 1.** Characteristics of the selected articles.

Author	Year	Sample size	Age	Sex	Race/Ethnicity	Study Design	Country	Population Definitions of at-risk	Intervention
McLeod et al^34^	2020	Total—429 Intervention—215 Control—214	Intervention 25-44 y 10 45-54 y 33 55-64 y 76 65-75 y 96 Control 25-44 y 14 45-54 y 40 55-64 y 71 65-75 y 89	Intervention Male 108 Female 107 Control Male 103 Female 111	Intervention Maori 32 Pacific 6 All others 177 Control Maori 33 Pacific 10 All others 171	Parallel-group two-arm single-blinded RCT	New Zealand	HbA1c 41-70 mmol/mol (5.9%-8.6%)	BetaMe: Mobile-device and web-based program with individual health coaching, educational resources, peer support forums, online goal tracking
Block et al^31^	2015	Total—339 Intervention—153 Control—176	Intervention Mean age (y) 52.0 Control Mean age (y) 54.0	Intervention Male 48 Female 52 Control Male 46 Female 54	Intervention White 109 Hispanic 7 Asian 41 Other 6 Control White 120 Hispanic 14 Asian 29 Other 13	RCT	America	BMI ⩾ 27 kg/m^2^, fasting glucose 5.55 to 6.94 mmol/L or HbA1c 39 to 46 mmol/mol, (5.7%-6.4%)	Alive-PD: Individualized websites, interactive emails, mobile app, automated phone calls, virtual teams
Fukuoka et al^30^	2015	Total—61 Intervention—30 Control—31	Intervention Mean age (y) 57.1 Control Mean age (y) 53.4	Intervention Male 23.3 Female 76.7 Control Male 22.6 Female 77.4	Intervention Asian 33.4 Black/African American 0.0 Hispanic/Latino 13.3 White (non-Hispanic) 43.3 Mixed race10.0 Control Asian 12.9 Black/African American 9.7 Hispanic/Latino 9.7 White(non-Hispanic) 61.3 Mixed race 6.4 s	RCT	America	BMI ⩾ 25, age ⩾ 35, diabetes test scores ⩾ 5, fasting plasma glucose of 100 to 125 mg/dL, HbA1c 57% to 7.0%, OGTT 12 to 200 mg/dL	Mobile-based Diabetes Prevention Program (mDPP): In-person lifestyle intervention sessions, interactive mobile app, pedometer
Fischer et al^29^	2016	Total—163 Intervention—82 Control—81	Intervention Mean age (y) 47.7 Control Mean age (y) 45.2	Intervention Male 23 Female 55 Control Male 15 Female 64	Intervention English speaking 27 Spanish speaking 51 Control English speaking 45 Spanish speaking 34	RCT	America	Adults with prediabetes (glycated hemoglobin (HbA1c) from 5.7% through 6.4% and BMI between 25 and 50 kg/m^2^	Text-message enhancement of DPP classes Optional individual motivational interviewing with a health coach via telephone
Ramachandran et al^38^	2013	Total—537 Intervention—271 Control—266	Intervention Mean age (y) 45.9 Control Mean age (y) 46.1	Men only study Intervention 271 Control 266	All participants were Indian men	prospective parallel-group RCT	India	Positive family history of T2DM or a BMI of 23 kg/m^2^	Text messages based on behavioral change tailored to participants Personalized education and motivation about a healthy lifestyle at baseline by a health coach
Nanditha et al^32^	2020	Total – 2062 (India [n = 1171], UK [n = 891]) Intervention—1031 Control—1031	Intervention Mean age (y) 52.1 Control Mean age (y) 52.0	Intervention Male 658 Female 373 Control Male 661 Female 370	Intervention Indians 584 UK residents 447 Control Indians 587 UK residents 444	RCT	UK and India	Prediabetes (upper range): HbA1c 6.0% to 6.4% (42-47 mmol/mol)	Text messages: educational, motivational, and supportive content on diet, physical activity, lifestyle, and smoking
Wong et al^36^	2013	Total—104 Intervention—54 Control—50	Intervention Mean age (y) 54.1 Control Mean age (y) 55.2	Intervention Male 49 Female 5 Control Male 48 Female 2	All participants were Chinese	single-blinded RCT	Hong Kong	Pre-diabetes within the last 3 mo: FBG 5.6 to 6.9 mmol/L or 2HPPG of 7.8 to 11.0 mmol/L after a 75 g glucose load	Text messages: educational information about diabetes, lifestyle modification and self-efficacy-enhancing techniques
Katula et al^33^	2022	Total—599 Intervention—299 Control—300	Intervention Mean age (y) 55.3 Control Mean age (y) 55.6	Intervention Male 115 Female 184 Control Male 116 Female 184	Intervention White 273 African American 16 Hispanic 7 Asian 4 Other 4 Control White 269 African American 23 Hispanic 12 Asian 2 Other 6	Single-blind RCT	America	HbA1c 5.7% to 6.4% (39-46 mmol/mol)	Digital DPP adapted from the Omada Health Program: Interactive behavioral change lessons, virtual peer group, health coach messages, tracked meals and physical activity with internet-accessible wearable devices
Fottrell et al^37^	2019	Total—12140 Intervention (mHealth)—4071 Other (PLA)—4021 Control—4048	Intervention 30-39 y 1329 40-49 y 1068 50-59 y 765 60-69 y 659 70-100 y 250 Control 30-39 y 1391 40-49 y 991 50-59 y 767 60-69 y 648 70-100 y 251	Intervention Male 1845 Female 2226 Control Male 1950 Female 2098	All participants were Bangladeshil	Three-arm, cluster RCT	Bangladesh	Intermediate hyperglycemia: FBG 5.5-6.8 mmol/L	Dmagic: mHealth mobile phone messaging promoting behavioral change

Abbreviations: BMI, body mass index; DPP, diabetes prevention program; FBG, fasting blood glucose; HbA1c, hemoglobin A1c; RCT, randomized controlled trial; T2DM, type 2 diabetes mellitus.

Eight (89%) out of 9 studies’ populations were of mixed gender^29-34,36,37^ with 1 study population being exclusively male.^35^ The 4 American studies defined an individual at risk of diabetes or in the prediabetes stage with HbA1c scores of 5.7% to 6.4% or 39 to 46 mmol/mol.^29-31,33^ Other studies recruited participants in the upper prediabetes range (HbA1c > 5.9%)^32^ or defined at-risk populations by BMIs greater than 23 kg/m^2^, positive family histories of type 2 diabetes mellitus (T2DM), oral glucose tolerance test scores (14-200 mg/dL) or fasting plasma glucose scores (100-125 mg/dL).^35^

Intervention methods varied across studies where differing combinations of health coaching, educational resources, peer support groups and goal tracking were mediated through various digital platforms.^29-37^ A single intervention^31^ used individually tailored websites and interactive emails while 7 interventions^29,30,32,33,35-37^ used mobile devices via text or voice messages,^29,32,33,35-37^ telephone-mediated coaching^29^ or mobile applications.^30,34^ Digital technology was supplementary to in-person or online coaching by a health coach in 5 studies^29,30,33,34,37^, four studies were automated and algorithm-driven.^31,32,35,36^

Table 2 summarizes the primary outcomes and results of the included studies. Three studies^31,33,34^ determined intervention efficacy by changes in HbA1c levels from baseline to the end of 12-month interventions with Block et al^31^ demonstrating significant reductions in HbA1c (−2.8 mmol/mol [95% CI −0.27, −0.24], *P* = .002) from a DPP mediated through individually tailored websites and interactive emails. Katula et al’s^33^ study demonstrated a shift in HbA1c levels from the prediabetes to the normal range (−0.23% [95% CI −0.26, −0.20]) due to an internet-accessible device (mobile, computer, smartwatch) mediated DPP, while McLeod et al’s^34^ mobile and web-based individual and peer support coaching intervention showed no difference between study arms.

**Table 2.** The primary outcomes of the reviewed randomized controlled trials.

Author	Duration	Measurement of the primary outcome	Intervention (mean changes ± SD)	Control (mean changes ± SD)	Risk ratio (95% CI) *P*-value
McLeod et al^34^	12 mo	HbA1c and weight (kg) change from baseline to 12-month follow-up	HbA1c: 0.0 mmol/mol Weight: −1.1 kg	HbA1c: 0.0 mmol/mol Weight: −0.7 kg	HbA1c: 95% CI −0.9, 0.9; *P* = .990, (intervention vs control) Weight: 95% CI −1.8, −0.5; *P* = .396 (intervention vs control)
Block et al^31^	12 mo	HbA1c and fasting glucose at 6 mo	HbA1c: −2.81 mmol/mol, −0.26% FBG: −0.41 mmol/L	HbA1c: −1.93 mmol/mol, −0.18% FBG: −0.21 mmol/L	HbA1c: 95% CI −0.27, 0.24; *P* = .002 FBG: 95% CI −0.44, −0.12; *P* < .001
Katula et al^33^	12 mo	Change in HbA1c from baseline to 12 mo	−0.23% (95% CI = −0.26, −0.20)	−0.16% (95% CI = −0.19, −0.12)	*P* = .001
Fukuoka et al^30^	5 mo	% Change in weight and BMI from baseline to 5-month follow-up	Change in weight of −6.2 ± 5.9 kg Change in weight (%) −6.8 ± 5.4 Change in BMI of−2.2 ± 2.2 kg/m^2^ Change in BMI (%) −6.6 ± 5.7	Change in weight of 0.3 ± 2.7 kg Change in weight (%) 0.3 ± 3.0 Change in BMI of 0.1 ± 1.0 kg/m^2^ Change in BMI (%) 0.3 ± 3.0	Weight change: 95% CI 4.6, 9.1; *P* < .001 BMI: *P* < .001
Fischer et al^29^	12 mo	Change in mean weight (lb)	−2.6 lb (95% CI -5.5 -0.2)	−0.6 lb (95% CI −2.7 to 1.6)	*P* = .05
Ramachandran et al^38^	24 mo	Incidence of T2DM	50 (18%)	73 (27%)	Hazard ratio 0·64, 95% CI 0.45, 0.92; P = 0·015
Nanditha et al^32^	24 mo	Incidence of T2DM: HbA1c ⩾ 6.5% (48 mmol/mol)	234 (22.7%)	216 (21.0%)	0.89; 95% CI 0.74, 1.07; *P* = .22
Wong et al^36^	36 mo	Incidence rate of DM over 12- and 24-mo period	12 mo: 3/54 (5.56%) 24 mo: 6/54 (11.11%)	12 mo: 8/50 (16%) 24 mo: 9/50 (18%)	12 mo: RR: 0.35, 95% CI 0.10, 1.24 24 mo: RR: 0.62, 95% CI 0.24, 1.61
Fottrell et al^37^	36 mo	combined prevalence of T2DM and intermediate hyperglycemia at the end of the intervention	Intermediate hyperglycemic: 315 (43.9%) Diabetes: 122 (17%) Combined: 437 (60.9%)	Intermediate hyperglycemic: 337 (47.3%) Diabetes: 126 (17.7%) Combined: 463 (65%)	0.99; 95% CI 0.70, 1.39; *P* = .941

Abbreviations: BMI, body mass index; DPP, diabetes prevention program; FBG, fasting blood glucose; HbA1c, hemoglobin A1c; RCT, randomized controlled trial; T2DM, type 2 diabetes mellitus; RR, risk ratio.

The primary outcome of the 2 studies^29,30^ was a percentage change in weight or BMI. Fukuoka et al’s^30^ mixed intervention with in-person DPP sessions, supplementary interactive mobile app and pedometer use proved effective in weight loss (6.8% [95% CI,4.6,9.1]) while Fischer et al^29^ proved the efficacy of text-message enhancement of DPPs in Spanish populations with a comparable portion of the study population losing 3% baseline weight (47.1% [95% CI 33.4-60.8]) compared to English speakers (20.6% [95% CI 7/0-34.2]).

Four studies^32,35-37^ examined an outcome of diabetes incidence in relation to DPP interventions mediated over mobile phone messaging with 3 studies^32,35,36^ yielding clinically significant results. Ramachandran et al^38^ and Wong et al’s^36^ studies examining behavioral change theory-oriented text messages both affected the lower cumulative incidence of T2DM in comparison to standard care (−18% [hazard ratio 0.64, 95% CI 0.45, 0.92; *P* = 015]; −11.11% [95% CI, 0.24, 1.61, *P* = .021]). Fortrell et al’s^37^ voice-messaging intervention and Nanditha et al’s^32^ contrastingly, showed no evidence of effect between intervention and control cohorts.

### Risk of bias

**Figure 2.**
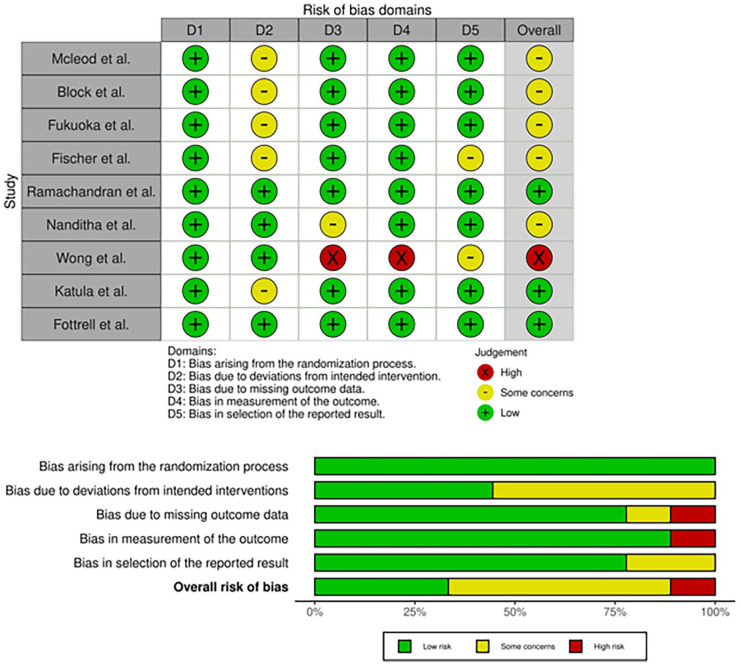
Risk of bias assessment for the various domains.

Using Cochrane Collaboration Tool’s RoB 2,^25^ Figure 2 details the result of assessment by domains for the selected studies. Four studies were identified with an overall low ROB,^29,33,35,37^ another with a high ROB due to attrition (incomplete follow-up and patient retention)^36^ and for 4 other studies, there were some concerns of attrition bias (patient non-compliance or retention) to a lesser degree.^30-32,34^ There was no selection bias in the reviewed studies due to the adequate randomization and allocation concealment in the studies. Regarding performance bias, by nature of the interventions, the majority of patients and caregivers could not be blinded but this did not affect study outcomes. Detection bias was present in 2 studies whose outcomes were not statistically significant^29,36^ and no reporting bias was identified as all measured outcomes were reported in the final analysis.

## Discussion

This review study identified 5 studies^30,31,35-37^ that showed clinically significant reductions of various diabetes risk related outcomes especially glycemic levels, weight, BMI and reduced incidence of T2DM, possibly strengthening available literature on the efficacy of 2 main digital modalities (computer and mobile devices) in diabetes prevention. Despite the statistical significance of these RCTs, the studies were significantly heterogeneous in their outcomes due to the large variation in the intervention methods. The study findings suggest that the efficacy of individual DPPs may be due to the synergistic effect of various modes of delivery (technological and in-person) rather than the superiority of one modality over the others. However, due to the small number of studies about each digital modality, the impact of these variations in the intervention could not be properly assessed.

In this study, there were 2 RCTs^31,33^ involving computer-based DPPs that yielded statistically significant results, proving the capacity of computers to fully automate DPPs or to act in a supplementary role – providing resources alongside in-person curriculums. For one study,^31^ after 6 months, the use of individualized websites and interactive emails improved the glycemic level, body weight and 8-year diabetes risk of people with prediabetes. The use of computer-mediated peer support groups, tracking and behavioral change curriculums yielded significant weight loss at 12 months,^33^ whereas mobile-mediated interventions led to positive outcomes such as greater weight loss over time and reduction in the risk of developing diabetes at 12 months.^30,35,36^ A text-messaging intervention where delivered content was individualised^39^ and one where messages were standardized to collective participant preferences,^36^ both decreased the cumulative incidence of T2DM. In agreement with previous evidence,^39,40^ supporting in-person DPP sessions with a mobile app for tracking progress and mediating educational resources achieved clinically significant weight loss,^30^ thus affirming the strength of mobile-mediated interventions in increasing patient adherence through positive reinforcement.^39,40^ This has also been linked to high retention rates in clinical trials.^29,30,32,33,35-37^

On the other hand, mobile-mediated DPPs are effective in consistently reinforcing patient education,^39,41^ self-monitoring and social support^39^ which prevents non-adherence. The high global levels of smartphone ownership^40^ further decrease economic expenditure and accommodate a widespread dissemination of intervention programs. Four text message-based programs focused on stage-based behavioral change were shown to significantly reduce the progression of prediabetes to diabetes with preventative outcomes of weight loss and diet logging.^29,35,38,42^ In examining a broad digitalization of DPPs, a 2017 meta-analysis correlates smartphone apps mediating health professional personal contact with increased weight loss, in comparison to complete automated contact.^43^ Evidently, by replacing in-person group exercise sessions with phone-app delivered home regimens, Fukuoka et al’s^30^ intervention yielded no change in glucose levels, supporting how retaining partial human factors in delivery is still imperative in patient adherence and response.

Regarding the effect of computer-based therapies in decreasing HbA1c in at-risk patients, the evidence is judged to be moderate in quality. The studies have a generally low risk of bias besides some concerns of attrition for participant retention.^31,33^ There is no great imprecision, indirectness, or publication bias but the heterogeneity of the intervention method and the data of statistical significance only being sourced from 3 studies, lowers confidence in the review’s results. For mobile-based therapies and their impact on reducing the incidence of T2DM, the evidence is judged to be of low quality. There is moderate to high risk in 2 out of 3 studies of statistical significance and the sample size in those studies was small (61 and 104 participants).^30,36^ The RCTs have no obvious imprecision, indirectness, or publication bias but the heterogeneity of intervention and study protocol were variable.

Digital health technology interventions play a pivotal role in augmenting existing strategies for the prevention of type 2 diabetes mellitus (T2DM). These interventions leverage innovative technological solutions to enhance outreach, education, monitoring, and management of individuals at risk for T2DM. By incorporating mobile applications, wearable devices, and online platforms, digital health technologies provide personalized and real-time interventions, promoting lifestyle modifications and behavior change. These tools facilitate continuous glucose monitoring, physical activity tracking, and dietary management, empowering individuals to take an active role in their health. Moreover, digital interventions enable healthcare providers to remotely monitor patients, offer timely if not real-time feedback, and tailor interventions based on individual progress. The integration of data analytics and machine learning further refines predictive models, identifying high-risk populations and customizing intervention strategies. In essence, digital health technology interventions not only complement but also transform conventional approaches to T2DM prevention, fostering a more dynamic, individualized, and proactive healthcare paradigm.

### Implications for practice

This study provided evidence for the generalized efficacy of health technologies in preventing diabetes by reducing risk-related outcomes as 5 studies within this review demonstrated statistically significant reductions in HbA1c and diabetes incidence. Further research is needed to translate this data into clinical settings. The feasibility of digitally mediated DPPs is supported by the gradual modernization of the healthcare system and public accessibility to technology but this can be better understood with more pragmatic trials involving diverse populations or interventions that are culturally adapted (which showed a positive outcome in one study.^29^ We also provided evidence of appropriateness, as diabetes prevention involves self-management and literature which identifies mobile-devices efficacy in increasing patient engagement^39,40^ and the high retention rates of studies within this review^29,30,32,33,35-37^ support this. Evidence of meaningfulness was unavailable as patient-rated outcomes were not examined in this review and effectiveness is difficult to determine with the moderate to low quality evidence of the chosen studies.

Establishing a sustainable infrastructure for digital diabetes prevention programs is imperative for its long-term success. This infrastructure should prioritize interoperability with existing health information systems, ensuring seamless data exchange and continuity of care. Robust cybersecurity measures, including secure data storage and transmission, are essential to protect patient information and comply with healthcare data protection regulations. The use of scalable, cloud-based architecture enables flexibility and accommodates a growing user base, while user-friendly interfaces cater to a diverse range of users. Integration with wearable and IoT devices allows for real-time data collection, and analytics tools provide insights for continuous program improvement. Training modules and support mechanisms ensure both healthcare providers and end-users can effectively utilize the program. Regulatory compliance frameworks, reliable telecommunication infrastructure, and continuous monitoring and maintenance further contribute to the infrastructure’s resilience. By addressing these components comprehensively, digital diabetes prevention programs can seamlessly integrate into healthcare systems, promoting sustained engagement and positive health outcomes.

### Limitations and future research

The following limitations should be considered when interpreting this data. First, there was a small number of studies that met the eligibility criteria in this systematic review and the heterogeneity of study design (population, intervention mode of delivery, method, duration) collectively led to difficulty in comparison and inability to perform a meta-analysis to assess the strength of data. Although we have restricted the studies to increase the robustness of our study, future studies might include other non-RCTs for a better understanding of the impact of DPPs. Second, the study populations in the included studies lacked diversity which could affect the scalability and accessibility of these interventions. The fact that some studies were single-gendered, others recruited from specific workplaces or ethnic groups without cultural adaptation of the DPP program, may have affected the participant engagement. Third, the study durations were short ranging from 5 to 36 months with the longest follow-up at 24 months and both these factors limit the external validity of DPP interventions which target a chronic and global health issue. Also, the reviewed studies were all written in English including the Hong Kong Study. This could potentially exclude crucial findings from studies not written in English. Articles not written in English from non-English speaking jurisdictions should also be reviewed in future studies.

In this study, we identified a research gap with studies that examined specific aspects of health technologies for increased patient engagement. Two studies imply the importance of partial human factors in interventions, attributing high retention rates to healthcare staff delivering the intervention^27^ and the participatory in-person activities.^37^ Therefore, studying the interactive nature of digital and in-person modalities may optimize intervention protocol. Understanding the difference between personalized or standardized interventions and full automation or partial digital mediation is important and can be further explored in future studies.

An examination of diverse digital health approaches across different populations, healthcare systems, and cultural contexts is needed to develop targeted and culturally sensitive digital intervention solutions. Also, research should focus on identifying determinants of successful implementation, such as user engagement, usability, and adherence, to optimize intervention designs for maximum impact. Thirdly, continuous evaluation of digital interventions over the long term is crucial to assess their effectiveness and sustainability, informing potential updates or modifications. Fourthly, exploring the integration of digital health technologies into existing healthcare systems, considering interoperability, data security, and impact on workflows, is essential for successful implementation on a broader scale. Lastly, investigating the cost-effectiveness and scalability of these interventions will be vital for their widespread adoption and integration into routine healthcare practices.

## Conclusion

In conclusion, this review supports the current literature’s generalized perspective on the efficacy of health technology in diabetes prevention and presents some evidence of positive correlations between digitally translated DPPs and improved diabetes risk-related outcomes. Yet, the heterogeneity in intervention protocol and study procedure and the lack of diverse populations or long-term data and follow-up undermine the possibilities of clinical effectiveness and the feasibility of public health implementation and scalability due to the risk of economic burden and the chance of patient non-adherence. Future research should include studies of larger diverse populations with longer duration and follow-up data and specific aspects of health technologies that increase patient engagement and the possibility of clinically significant outcomes.

## Supplemental Material

sj-docx-1-end-10.1177_11795514241246419 – Supplemental material for The Role of Digital Health Technology Interventions in the Prevention of Type 2 Diabetes Mellitus: A Systematic Review Supplemental material, sj-docx-1-end-10.1177_11795514241246419 for The Role of Digital Health Technology Interventions in the Prevention of Type 2 Diabetes Mellitus: A Systematic Review by Vivien Nguyen, Paige Ara, David Simmons and Uchechukwu Levi Osuagwu in Clinical Medicine Insights: Endocrinology and Diabetes

## Declarations

**Ethics approval and consent to participate:** Ethics was not required, however, the study protocol was registered with the International Prospective Register of Systematic Reviews PROSPERO (#: CRD42022344100).

**Consent for publication:** Not applicable

**Availability of data and materials:** Data for this study has been presented in the text.
